# Beyond pain intensity: coping, social disconnection, and depression among Black older adults

**DOI:** 10.3389/fpain.2026.1959380

**Published:** 2026-10-21

**Authors:** Sheria G. Robinson-Lane, Patricia Tillman-Meakins, Mary R. Janevic

**Affiliations:** 1 Department of Systems, Populations, and Leadership, University of Michigan School of Nursing, Ann Arbor, MI, United States; 2 Department of Health Behavior and Health Equity, University of Michigan School of Public Health, Ann Arbor, MI, United States

**Keywords:** African American, coping skills, interpersonal relations, loneliness, pain management, psychological distress

## Abstract

**Background:**

Pain is a physiological, psychological, and social stressor that contributes to functional disability and depression. Among Black older adults who often face multiple stressors, pain management may be deprioritized, increasing the risk of pain interference and exacerbation of depressive symptoms. The Roy Adaptation Model provides a multidimensional framework for understanding these relationships and identifying targets for culturally responsive interventions. This study examined the relationships between pain interference, adaptive coping, and social disconnection with depressive symptoms among a cohort of Black older adults with chronic pain.

**Methods:**

The primary analysis included baseline survey data from 299 Black participants with complete data who were enrolled in a chronic pain self-management program. Measures included the PROMIS Emotional Distress-Depression 4a scale, the PROMIS Pain Interference 6a scale, the Coping and Adaptation Processing Scale (CAPS), and the UCLA Loneliness Scale. Multiple linear regression examined the relationships of pain interference, adaptive coping, and social disconnection with depressive symptoms, adjusting for age and gender. Analysis examined differences by living arrangement and added living alone versus with others to the regression models.

**Results:**

The primary model was significant, F(5, 293) = 36.74, *p* < 0.001, explaining 39.2% of the variance in depressive symptoms. Social disconnection was the strongest predictor (*b* = 1.90, *β* = 0.389, *p* < 0.001), followed by adaptive coping (*b* = −0.27, *β* = −.190, *p* = 0.001) and pain interference (*b* = 0.20, *β* = 0.180, *p* = 0.002). Participants living alone reported greater social disconnection than those living with others, although unadjusted depressive symptoms did not differ. In the expanded model, living alone was associated with lower depressive symptoms after adjustment for social disconnection and other covariates (b = −1.77, *β* = −.104, *p* = 0.024).

**Discussion:**

Findings, though specific to this cohort, support a multidimensional interpretation of chronic pain encompassing the physiologic, interdependence, and self-concept modes of the Roy Adaptation Model. Wholistic pain assessment may benefit from extending beyond pain intensity to include functional interference, coping capacity, depressive symptoms, and social relationship quality. Culturally responsive interventions may strengthen adaptive coping strategies and improve meaningful social connections while addressing pain-related functional limitations.

## Introduction

Chronic pain and depression are often inextricably tied together and commonly referred to as the pain-depression cycle (1–5). A recent review of 376 studies across 50 countries found that approximately 40% of adult participants with chronic pain also reported depressive symptoms (6). The exact mechanisms of the bidirectional relationship between pain and depression are unclear. However, what is known is that chronic pain is a multidimensional experience. It is influenced by cultural context and can affect individuals physically, emotionally, spiritually, and socially—ultimately having a negative effect on quality of life (7–11). This is a significant shift from the historically biomedically focused views of pain that center and prioritize physical sensation and related pathophysiology over the psychosocial and cognitive dimensions of pain. A growing body of evidence has begun to demonstrate that contextual factors such as racism, discrimination, stigma, and poverty often shape both pain experiences and incidence of depressive symptoms—occurrences that are commonly experienced among Black older adults (12–14). Interestingly, although Black adults often report high levels of pain intensity and pain interference (15–17) their reported depressive symptoms and rates of diagnosed depression remain comparatively lower than other ethnoracial groups (18, 19). Conventional depression screening measures may not fully capture culturally patterned expressions of psychological distress among Black adults, potentially contributing to underrecognition of depressive symptoms (20). Nevertheless, this apparent paradox raises important questions about the factors that may influence depressive symptoms among Black older adults with chronic pain and whether existing models of pain mechanisms adequately explain the relationships between pain and depression. The present study examines a cohort of Black older adults with chronic pain and assesses the factors that may be contributing to variation in depressive symptoms. Understanding these relationships requires consideration of how pain theories have evolved and whether adaptation may help to explain variability in pain-depression outcomes.

### Evolving theories of pain

Historically, pain was viewed primarily as a physiological response to bodily injury. One of the earliest mechanistic explanations, Specificity Theory, posited that pain was a distinct sensory experience transmitted through specialized receptors and neural pathways (21, 22). Early research grounded in this theory examined how different types of skin stimuli—including warmth, cold, and pressure—produced varied responses that traveled through peripheral nerves and the spinal cord to the brain (23). Intensity Theory later challenged the premise that pain relied exclusively on pain pathways. Through his work on nerve stimulation, neurologist Wilhelm Erb determined that any stimulus at the right intensity could cause pain along any sensory pathway, including those caused by disorders of the nerves (24). Earlier work by physician Erasmus Darwin similarly described pain as an accumulation of “sensorial power,” suggesting that at high enough levels, pain responses bypassed the conscious thoughts of the mind and became more instinctual (P.275) (25). These perspectives contributed to an emerging distinction between the physiological transmission of sensory information and the mind's perception and interpretation of that information.

The perceptual dimensions of the pain experience became more pronounced in the work of experimental psychologist and physiologist John Paul Nafe. Nafe argued that intense stimulation resulting in pain was almost always accompanied by a conscious experience that influenced subsequent pain events in patterns that were likely both physiological and psychological (26). These ideas contributed to the development of the more physiologically focused Pattern Theory, which used electrical current visualization and measurement devices [e.g., cathode-ray oscilloscope (CRO), galvanometer] to demonstrate how various patterns and intensities of neural activity cause pain rather than single specialized pathways (22). Ronald Melzack and Patrick Wall further advanced understanding of pain through the Gate Control Theory, which reestablished the importance of psychological states, emotions, and previous pain experiences in modulating pain responses (27). The Gate Control Theory proposed that pain responses are influenced by various physiological and psychological inputs within the nervous system and positioned pain perception as an active process involving multiple regions of the brain rather than simple responses to peripheral stimulation (27). Melzack's later work, Neuromatrix Theory, focused more exclusively on pain as a multidimensional experience influenced by genetics, sensory inputs, cognitive and emotional processes, and individual stress responses (28). Collectively, these theories gave rise to contemporary understandings of pain as individualized experiences shaped by interacting biological, psychological, and social factors.

Current conceptualizations therefore increasingly recognize that stress appraisal, coping processes, and social-relational experiences may influence not only pain perception, but also pain interference, emotional well-being, and functional outcomes (29–32). Within this broader theoretical progression, models of stress and adaptation may offer additional insight into why individuals who are experiencing similar levels of chronic pain may report differing psychological and functional outcomes. The Roy Adaptation Model provides a framework for examining how pain-related demands, coping processes, and responses across psychological, functional, and interpersonal domains may contribute to variation in depressive symptoms.

### Pain and the roy adaptation model

The Roy Adaptation Model is a reciprocal interactive change process model developed by Sister Callista Roy (33). The model describes how individuals respond to focal, contextual, and residual stimuli through coping processes aimed at maintaining or improving health and function (34). The resulting responses are expressed across four domains, or adaptive modes: physiologic, self-concept, role function, and interdependence. Responses fall within a continuum ranging from adaptive, supporting integrity and well-being, to ineffective or not adequately addressing stressors. In the physiologic mode, adaptive behaviors are focused on restoration of body homeostasis through appropriate oxygenation, nutrition, elimination, activity, rest, and/ or protection. Self-concept mode adaptive behaviors emphasize positive beliefs and feelings an individual has about themselves, including their sense of self-organization, their moral and ethical compass, and their ideal self. Adaptive behaviors in the role function domain lead to positive perceptions about oneself and their responsibilities in relation to others. Finally, adaptive behaviors in the interdependence mode are focused on improving secure relationships and developing or maintaining adequate support systems. Effective use of the model engages assessment to identify adaptive or deficient responses, and the related stimuli, in order to develop appropriate interventions aimed at improving related health outcomes. As humans are bio-psycho-social-spiritual beings, adaptive responses are usually required across all domains. This is particularly true for a pain-related stimulus that has become chronic.

Acute pain stimulates the sympathetic nervous system and accompanying fight-or-flight adaptive behaviors such as tachypnea, tachycardia, diaphoresis, and other physiologic responses. Acute pain, within the Roy model, would largely be considered a focal stimulus, as it requires immediate attention for remediation. The primary goal of the person experiencing acute pain is body homeostasis. In the context of chronic pain, or pain lasting longer than three months, pain may remain a focal stimulus when it demands immediate attention, or it may operate as a contextual stimulus that continues to shape daily functioning and responses to other life demands (35–41). Pain interference reflects the extent to which persistent pain disrupts activities, social interactions, sleep, enjoyment of life, and the fulfillment of one's typical roles (42). Adaptive coping reflects the cognitive and behavioral processes individuals use to manage these demands. Perceived social disconnection, reflected by loneliness, represents an ineffective response within the interdependence mode, and depressive symptoms may be conceptualized primarily as a psychological outcome associated with adaptation in the self-concept mode. Therefore, the Roy Adaptation Model provides a useful framework for considering how functional, coping, and interpersonal factors may be associated with emotional outcomes among individuals experiencing chronic pain.

## Methods

The Roy Adaptation Model informed the selection and organization of the study variables. Chronic pain was conceptualized as the focal stimulus, pain interference and social disconnection as responses within the physiologic and interdependence modes, respectively, and adaptive coping as a process operating across adaptive modes (see Figure 1). Depressive symptoms were conceptualized as an outcome reflecting compromised adaptation. We hypothesized that greater pain interference and social disconnection, along with lower adaptive coping, would be associated with greater depressive symptoms.

**Figure 1:**
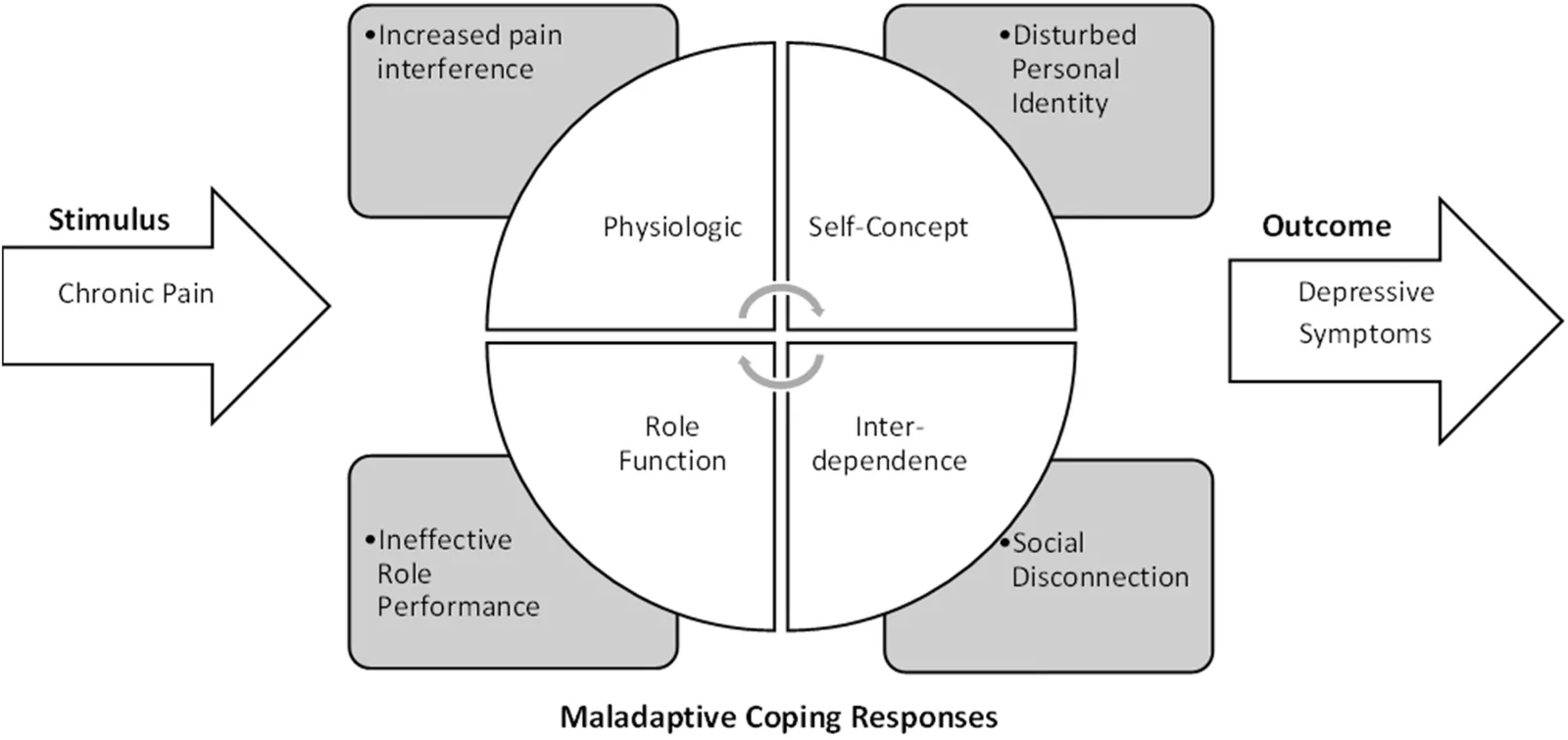
Chronic pain and depression within the roy adaptation model.

### Study design

The present secondary analysis included 335 participants who identified as Black from the larger STEPS randomized controlled trial (NCT05278234)—a study examining a community health worker-led chronic pain self-management intervention compared to usual care (43). Information on sample size and power calculation, participant recruitment, and other details of the primary study are reported elsewhere (43). Study participants were urban, community-dwelling, Black older adults (age 50 years or older) who self-reported muscle or joint pain on most days for three months or longer, rated at an average of 4 or greater (0–10 scale) in the past week, that interfered with usual activities one or more days in the past month. Age 50 was used as the lower-end age cutoff to be inclusive of all senior housing residents interested in study participation and to account for accelerated aging and earlier onset of age-related health declines observed in Black adults consistent with the weathering hypothesis (44). Exclusion criteria included serious or acute illness, hospitalization in the past 30 days, or other psychiatric or cognitive disorders that would prevent study participation. Participants were recruited at local senior living and community centers, through social media advertisements, and through a local partnering health care organization. All participants provided written consent, and the study was approved by the University of Michigan Institutional Review Board (HUM00206235).

### Measures

***Pain interference***. Pain interference was evaluated using the 6-item subscale of the Patient-Reported Outcomes Measurement Information System (PROMIS)-43 Adult Profile (41). Likert scales ranging from 1 (not at all) to 5 (very much) were used for participants to self-evaluate how much pain has interfered with daily activities such as household chores, social activities, sleep, and enjoyment of life over the past 7 days. Standardized T-Scores are used and referenced to the U.S. general population (mean 50, SD 10). The PROMIS Pain Interference short form has demonstrated strong \ measurement precision and close correspondence with the full item bank (42).
***Depressive symptoms.*** Depressive symptoms were examined using the PROMIS Emotional Distress-Depression Short Form 4a. Participants noted feelings of worthlessness, helplessness, depression, and hopelessness over the past 7 days using Likert scales ranging from 1 (never) to 5 (always). Internal consistency ranged from 0.93 to 0.95 and reliability ranged from 0.8 to 0.86 (45). Standardized T-Scores are used and referenced to the U.S. general population (mean 50, SD 10).
***Adaptive coping.*** The Coping and Adaptation Processing Scale (CAPS) examines cognitive and behavioral coping strategies through 15 questions about typical responses to a crisis or extremely difficult event (46). Scoring per item ranges from 1 (never) to 4 (always). The reliability of the tool has been noted at 0.82 (46).
***Social disconnection.*** The 3-item UCLA Loneliness Scale was used to assess subjective relational feelings of social disconnection, including a lack of companionship, feeling left out, and feeling isolated (47). Participants rated how often they experienced each feeling using response options ranging from 1 (hardly ever) to 3 (often). Scale reliability was noted at 0.72.

## Data analysis

Descriptive statistics were calculated to summarize participant characteristics and study variables. Bivariate associations among depressive symptoms, pain interference, adaptive coping, and social disconnection were examined using Pearson correlation coefficients. Multiple linear regression was then conducted among participants with complete data (*N* = 299) to examine the independent associations of pain interference, adaptive coping, and social disconnection with depressive symptoms. PROMIS depression T-scores served as the dependent variable, and age and gender were included as covariates. All predictors were entered simultaneously. A secondary analysis was conducted among female participants with no missing data (*N* = 264, or 86.6% of analytic sample) to assess whether the observed associations were consistent within the largest gender subgroup. One participant reporting a gender other than male or female was excluded from analyses adjusting for gender.

Additional analysis examined living arrangements as a contextual indicator of participants’ social environments. Participants who reported that no other members lived in their household were categorized as living alone and those reporting one or more additional household members were categorized as living with others. Independent-samples t tests were used to compare perceived social disconnection and depressive symptoms by living arrangement. Living arrangement was then added to the full-sample and female-only regression models to determine if it was independently associated with depressive symptoms after accounting for perceived social disconnection and the other study variables. Regression assumptions were evaluated by examining residual plots, tests of residual normality, variance inflation factors, and the Breusch-Pagan/Cook-Weisberg test. As the latter indicated nonconstant error variance, regression models were estimated using heteroskedasticity-robust standard errors. Statistical significance was established at *p* < 0.05, and analyses were conducted using STATA version 19 (48).

## Results

Participants reported moderate pain intensity rated at 6.41 (SD 1.98) on a 0–10 rating scale and pain interference one standard deviation greater than the US population average (61.14, SD 7.63). The overall level of depressive symptoms was average at 48.63 (SD 8.41). Other demographic characteristics are noted in Table 1. Bivariate correlations among depressive symptoms, pain interference, adaptive coping, social disconnection, and age are presented in Table 2. All correlations were statistically significant (*p* < 0.001), with social disconnection showing the strongest correlation with depressive symptoms (r = 0.561), followed by pain interference (r = 0.429) and adaptive coping (r = −0.403). The primary multiple linear regression model was statistically significant, F(5, 293) = 36.74, *p* < 0.001, and explained 39.2% of the variance in depressive symptoms (adjusted R^2^ = 0.382; see Table 3). Social disconnection was the strongest independent predictor of greater depressive symptoms (b = 1.90, 95% CI [1.30, 2.50], *β* = 0.389, *p* < 0.001). Greater pain interference was also associated with higher depressive symptoms (b = 0.20, 95% CI [0.07, 0.32], *β* = 0.180, *p* = 0.002), whereas greater adaptive coping was associated with lower depressive symptoms (b = −0.27, 95% CI [−0.43, −0.12], *β* = −0.190, *p* = 0.001). Age was not significantly associated with depressive symptoms (b = −0.08, 95% CI [−0.19, 0.02], *β* = −0.081, *p* = 0.101). The omnibus test for gender was also not statistically significant, F(1, 293) = 0.02, *p* = 0.898.

**Table 1:** Participant characteristics and study variables (*N* = 335).

Variables	Mean (SD) or *N* (%)
Age, years	67.03 (8.01)
Gender	
Male	44 (13.1%)
Female	290 (86.6%)
Other	1 (0.3%)
Education level	
Less than high school	15 (4.5%)
High school graduate or GED	34 (10.1%)
Vocational/technical	18 (5.4%)
Some college	98 (29.3%)
Associate degree	55 (16.4%)
Bachelor's degree	49 (14.6%)
Graduate degree	57 (17.0%)
PhD	5 (1.5%)
Employment status	
Employed full time	34 (10.1%)
Employed part-time	18 (5.4%)
Retired	198 (59.1%)
Unemployed	49 (14.6%)
Other	33 (9.9%)
Living arrangement	
Lives alone	148 (44.6%)
Lives with others	184 (55.4%)
Household size, persons	1.94 (1.17)
Depressive symptoms	48.76 (8.43)
Pain intensity (0–10 scale)	6.41 (1.98)
Pain interference	61.01 (7.54)
Adaptive coping	48.47 (5.83)
Social disconnection	4.68 (1.74)

Continuous variables are presented as M (SD); categorical variables are presented as *n* (%). Sample sizes for individual variables vary due to missing data. Missing data ranged from 0% to 6.3%.

**Table 2:** Descriptive statistics and correlations among study variables.

Variable	M	SD	1	2	3	4	5
1. Depressive Symptoms	48.63	8.41	–				
2. Pain Interference	61.14	7.63	0.429*	–			
3. Adaptive Coping	48.38	5.86	−0.403*	−0.277*	–		
4. Social Disconnection	4.66	1.72	0.561*	0.456*	−0.378*	–	
5. Age	67.10	8.01	−0.246*	−0.229*	0.203*	−0.216*	–

*N* = 299. Values are Pearson correlation coefficients.

*p < 0.001.

**Table 3:** Multiple linear regression predicting depressive symptoms.

Predictor		Black Sample (*n* = 299)		Black Female Sample (*n* = 264)		
	*b*	95% CI	*β*	*b*	95% CI	β
Age	−0.08	[−0.19, 0.02]	−0.081	−0.08	[−0.18, 0.03]	−0.073
Gender						
Female	−0.15	[−2.46, 2.16]	−0.006	-	-	-
Pain Interference	0.20	[0.07, 0.32]	0.180	0.17	[0.04, 0.30]	0.155
Adaptive Coping	−0.27	[−0.43, −0.12]	−0.190	−0.27	[−0.44, −0.11]	−0.193
Social Disconnection	1.90	[1.30, 2.50]	0.389	1.85	[1.19, 2.52]	0.385
*R^2^*		.392			.369	
Adjusted R^2^		.382			.359	
*F*(df)		36.74 (5, 293)			37.43 (4, 259)	
Model *p*		<0.001			<0.001	

Confidence intervals and significance tests are based on heteroskedasticity-robust standard errors. Male is the gender reference category.

The secondary analysis among female participants produced a similar pattern of findings. The model was statistically significant, F(4, 259) = 37.43, *p* < 0.001, and explained 36.9% of the variance in depressive symptoms (adjusted R^2^ = 0.359). Social disconnection remained the strongest independent predictor of depressive symptoms (b = 1.85, 95% CI [1.19, 2.52], *β* = 0.385, *p* < 0.001). Greater pain interference continued to be associated with higher depressive symptoms (b = 0.17, 95% CI [0.04, 0.30], *β* = 0.155, *p* = 0.013), and adaptive coping was associated with lower depressive symptoms (b = −0.27, 95% CI [−0.44, −0.11], *β* = −0.193, *p* = 0.001). Age was not significantly associated with depressive symptoms among the female participants (b = −0.08, 95% CI [−0.18, 0.03], *β* = −0.073, *p* = 0.171).

Living arrangement was associated with social disconnection but not with depressive symptoms in the unadjusted analyses. Participants living alone reported greater social disconnection (M = 4.91, SD = 1.81) than participants living with others (M = 4.52, SD = 1.66), t(330) = −2.04, *p* = 0.043. In contrast, mean depressive symptom scores did not differ significantly between participants living alone (M = 48.32, SD = 8.07) and those living with others (M = 49.05, SD = 8.67), t(330) = 0.79, *p* = 0.429. In the expanded full-sample model (*N* = 298), living alone was associated with lower depressive symptom scores after adjustment for age, gender, pain interference, adaptive coping, and social disconnection (b = −1.77, 95% CI [−3.30, −0.24], *β* = −0.104, *p* = 0.024). The model explained 40.4% of the variance in depressive symptoms (adjusted R^2^ = 0.392). A similar association was observed among female participants (b = −1.73, 95% CI [−3.35, −0.10], *β* = −0.104, *p* = 0.037). Across both expanded models, social disconnection remained the strongest predictor of depressive symptoms.

## Discussion

This study aimed to use the Roy Adaptation Model to clarify the relationship between pain and depression by examining the associations of pain interference, adaptive coping, and social disconnection with depressive symptoms among Black older adults with moderate to severe chronic pain. Social disconnection was the strongest independent correlate of depressive symptoms, followed by adaptive coping and pain interference. Greater pain interference and social disconnection were associated with higher depressive symptoms, while adaptive coping was associated with fewer symptoms. This pattern was consistent among female participants, the largest subgroup. Altogether, the findings suggest that chronic pain among this cohort of Black older adults reflects the combined influence of functional limitations, coping processes, and social environments (see Figure 1). The findings support a multidimensional framework of chronic pain that encompasses the physiologic and interdependence modes of the Roy Adaptation Model and suggest a potential role for the self-concept mode. Future studies that incorporate measures of personal identity and self-efficacy in relational role performance may provide important insights into how adaptation across the self-concept and role-function domains contributes to more integrated adaptation and lower risk for depressive symptoms among adults with chronic pain.

Chronic pain may function as a focal stimulus within the Roy framework, requiring ongoing physiological, psychological, and social adaptation. Pain interference reflects the extent to which pain disrupts daily life and may therefore affect multiple adaptive modes. Limitations in mobility, work, family responsibilities, social participation, and other activities critical to quality of life may affect individuals’ capacity to fulfill valued roles, maintain meaningful relationships, and preserve a positive sense of self. The association between pain interference and depressive symptoms suggests that understanding the consequences of pain for daily functioning may be as important as assessing its intensity.

The inverse relationship between adaptive coping and depressive symptoms further highlights the importance of coping processes in adaptation to chronic pain. Within the Roy Adaptation Model, coping processes shape how individuals interpret and respond to focal and contextual stimuli and whether their responses promote or disrupt adaptation. Adaptive coping may support emotional regulation, problem-solving, confidence, and a sense of control despite persistent pain. In previous work examining responses to multiple stressors among Black older adults, supportive environments including faith communities and local health agencies, and indicators of adaptive capacity such as emotional regulation and role adjustment, were identified as important resilience resources (49). These findings provide context for considering adaptive coping as one process that may contribute to resilience over time; however, resilience was not directly assessed in the present study, and the current findings are therefore interpreted in terms of coping and adaptation rather than resilience.

Consistent with this framing, lower psychological distress has been associated with adaptive coping strategies across the physiologic, interdependence, role function, and self-concept modes of the Roy Model, including exercise and assistive device use, supportive relationships with family and friends, active community engagement, prayer, meditation, and perseverance (50).

Resilience should not be conceptualized as a cultural characteristic or an expectation that Black older adults endure burdensome pain without adequate support. Rather, resilience emerges from interactions among personal coping capacities, social relationships, culturally salient resources, community environments, and access to appropriate healthcare and social services (51–53). Adaptive coping may represent one component of this broader process; however, the present analysis does not explain how wider social and structural conditions shape adaptation. Clinical assessment may therefore benefit from attention to both the coping resources individuals use and the unmet needs that may exceed those resources.

The strong association between social disconnection and depressive symptoms emphasizes the importance of the interdependence mode. Within the Roy Adaptation Model, interdependence involves the giving and receiving of love, respect, support, and meaningful connection. Chronic pain may disrupt this mode by limiting social participation, increasing dependence on others, altering family relationships, or reducing opportunities for reciprocal engagement. At the same time, limited or unsatisfying relationships may deprive individuals of emotional affirmation, instrumental assistance, and encouragement that could support adaptation to pain. Social disconnection should therefore be viewed as an integral component of the pain experience rather than as a peripheral social characteristic.

The living-arrangement findings further demonstrate that household composition and social connection are related but distinct. Participants living alone reported greater social disconnection than those living with others, yet depressive symptoms did not differ by living arrangement in the unadjusted analysis. After social disconnection and other study variables were considered, living alone was associated with modestly lower depressive symptom scores. This finding does not suggest that living alone is protective against depression. Instead, it indicates that living alone does not necessarily represent ineffective interdependence, just as living with others does not guarantee supportive or emotionally satisfying relationships. Some older adults who live alone may experience autonomy and strong connections outside the household, whereas those sharing a home may experience conflict, caregiving strain, limited reciprocity, or emotional isolation. Assessments should therefore examine the quality, availability, and meaning of relationships rather than relying on household size as a proxy for social support.

The findings have important implications for wholistic pain assessment, or assessment of the whole person across physical, psychological, and social dimensions. Traditional pain evaluations often emphasize pain intensity, location, and medication use, with less attention to how pain affects identity, coping confidence, social participation, and role disruption. A more comprehensive assessment may include pain interference, depressive symptoms, coping capacity, social disconnection, role functioning, and access to meaningful support. Clinicians should explore whether individuals can continue valued activities, how pain has affected family and community roles, which coping strategies remain effective, and whether existing relationships provide the desired support. Such assessments may identify needs that are not captured by pain intensity ratings alone.

Wholistic assessment is particularly important for Black older adults, whose pain experiences may be shaped by cumulative exposure to racism, unequal treatment within healthcare, economic disadvantage, caregiving expectations, and limited access to culturally responsive services. These contextual stimuli may influence pain expression, help-seeking, treatment preferences, and trust in clinicians. These same factors may also shape how depressive symptoms are expressed and whether or not they are recognized and addressed in clinical care. At the same time, extended family networks, faith traditions, spirituality, community participation, collective responsibility, and perseverance may serve as important cultural resources. Culturally responsive pain care should recognize these strengths without assuming that all Black older adults use or benefit from the same coping strategies. Clinicians should ask individuals which cultural, relational, and spiritual resources are meaningful to them and how those resources might be incorporated into care.

Interventions informed by the Roy Adaptation Model should address multiple adaptive modes rather than focusing solely on symptom reduction. Physiologic strategies may include appropriate pain treatment, exercise, rehabilitation, sleep support, and assistive devices. Self-concept approaches may strengthen coping confidence, emotional regulation, positive identity, and the ability to manage depressive symptoms. Role-function interventions may help individuals modify activities, renegotiate responsibilities, and maintain valued family and community roles. Interdependence-focused strategies may promote meaningful social participation, strengthen supportive relationships, reduce loneliness, and connect individuals with peer, community, or faith-based resources. Community health workers, peer facilitators, faith communities, and culturally concordant group programs may be particularly well positioned to integrate these domains while acknowledging the cultural and structural contexts in which pain is experienced.

### Limitations

Several limitations should be considered. First, the cross-sectional design does not establish the direction or causal sequence of the associations among pain interference, adaptive coping, social disconnection, living arrangement, and depressive symptoms. Depressive symptoms may influence perceptions of pain interference, coping capacity, or social connection, and these relationships are likely reciprocal. Further, the household measure identified how many other people lived in the home but did not capture relationship quality, household composition, conflict, caregiving responsibilities, or whether living alone was voluntary. The inverse adjusted association between living alone and depressive symptoms should therefore be interpreted cautiously. Additional limitations include the primary female sample, reliance on self-report measures, complete-case analysis with variable-level missingness up to 6.3% and recruitment from a chronic pain self-management trial, which may limit generalizability to Black older adults who do not enroll in such programs. Further, although the focus on Black older adults in this study addresses an important gap, the absence of a racial or ethnic comparison group means that the findings should not be interpreted as unique to Black older adults. Overall, this study's findings are specific to the included cohort and should not be generalized to represent all Black older adults. Future longitudinal and qualitative research should examine how adaptation changes over time and explore the cultural, relational, and structural processes underlying these associations.

## Conclusion

Despite these limitations, the findings demonstrate the value of using the Roy Adaptation Model to understand chronic pain as a multidimensional experience. Pain interference, adaptive coping, and social disconnection were each independently associated with depressive symptoms, with social disconnection emerging as the strongest correlate. The findings support moving beyond assessments centered primarily on pain intensity toward approaches that examine how older adults function, cope, maintain valued identities and roles, and connect with others. Identifying both adaptive strengths and areas of ineffective adaptation may inform culturally responsive interventions designed to support emotional well-being, meaningful social connection, and quality of life among Black older adults living with chronic pain.

## Data Availability

The raw data supporting the conclusions of this article will be made available by the authors, without undue reservation.
